# Targeting CD82/KAI1 for Precision Therapeutics in Surmounting Metastatic Potential in Breast Cancer

**DOI:** 10.3390/cancers13174486

**Published:** 2021-09-06

**Authors:** Maximillian Viera, George Wai Cheong Yip, Han-Ming Shen, Gyeong Hun Baeg, Boon Huat Bay

**Affiliations:** 1 Department of Anatomy, Yong Loo Lin School of Medicine, National University of Singapore, Singapore 117594, Singapore; antmax@nus.edu.sg (M.V.); georgeyip@nus.edu.sg (G.W.C.Y.); 2 Department of Physiology, Yong Loo Lin School of Medicine, National University of Singapore, Singapore 117593, Singapore; hmshen@um.edu.mo; 3 Faculty of Health Sciences, University of Macau, Taipa, China; 4 Ciechanover Institute of Precision and Regenerative Medicine, School of Life and Health Sciences, Chinese University of Hong Kong, Shenzhen 518172, China

**Keywords:** metastasis suppressor, tetraspanins, tyrosine kinase inhibitors, etoposide, CD82 mimics, epigenetic drugs

## Abstract

**Simple Summary:**

Breast cancer-related deaths are mainly due to the spread of cancer cells to distant organs (a process termed metastasis). CD82, also known as KAI1, is an established metastasis suppressor that has been documented to be lowly expressed in metastatic breast cancer. Hence, CD82 could possibly be a feasible molecular target for impeding metastases in breast cancer patients. Here, we propose a precision oncology-based model of preventing metastases by an appropriate selection of non-metastatic breast cancer patients with low CD82 expression. Potential therapeutic options for restoring CD82 levels that could be administered include the repurposing of existing chemotherapeutic drugs such as tyrosine kinase inhibitors and etoposide, as well as the use of CD82 peptide mimics and non-coding RNA-based therapeutics.

**Abstract:**

Metastasis is the main cause of mortality in breast cancer patients. There is an unmet need to develop therapies that can impede metastatic spread. Precision oncology has shown great promise for the treatment of cancers, as the therapeutic approach is tailored to a specific group of patients who are likely to benefit from the treatment, rather than the traditional approach of “one size fits all”. CD82, also known as KAI1, a glycoprotein belonging to the tetraspanin family and an established metastasis suppressor, could potentially be exploited to hinder metastases in breast cancer. This review explores the prospect of targeting CD82 as an innovative therapeutic approach in precision medicine for breast cancer patients, with the goal of preventing cancer progression and metastasis. Such an approach would entail the selection of a subset of breast cancer patients with low levels of CD82, and instituting an appropriate treatment scheme tailored towards restoring the levels of CD82 in this group of patients. Proposed precision treatment regimens include current modalities of treating breast cancer, in combination with either clinically approved drugs that could restore the levels of CD82, CD82 peptide mimics or non-coding RNA-based therapeutics.

## 1. Introduction

Approximately 2.26 million new female breast cancer cases were diagnosed globally in 2020 [1]. Furthermore, it has been estimated that 20–30% breast cancer patients treated at the primary tumor stage, will spread to other organs in the body [2,3]. Hence, there is an urgent need to develop more effective treatment strategies for combating breast cancer, in particular, hindering breast cancer spread to distant sites such as the lungs and brain, as metastasis is known to be the leading cause of cancer-related deaths [2,3,4,5].

Metastasis refers to the spread of cancer cells originating from a primary tumor, travelling through the bloodstream or lymphatic system, to form new tumors at a distant site in the body. The metastatic process is complex and involves many steps and, hence, it is an uphill task to elucidate the mechanisms involved in the different steps of metastasis. Traditionally, the concept for metastasis was believed to be the end-point of cancer progression, with the view that metastases only developed at the late stages of cancer, at which point the cancer is deemed incurable [6]. In the linear progression model, heterogeneous clones undergo successive mutations and selection before acquiring metastatic properties [3,7]. However, there have been reports indicating that cancer cells tend to show a ‘metastatic phenotype’ from the onset when the tumor is still small [8,9], as illustrated in the parallel progression model [3,10]. Notably, these studies revealed that primary tumor cells and metastatic cells showed similar gene expression patterns, implying that the propensity of a tumor to metastasize is determined early during cancer development.

**Figure 1:**
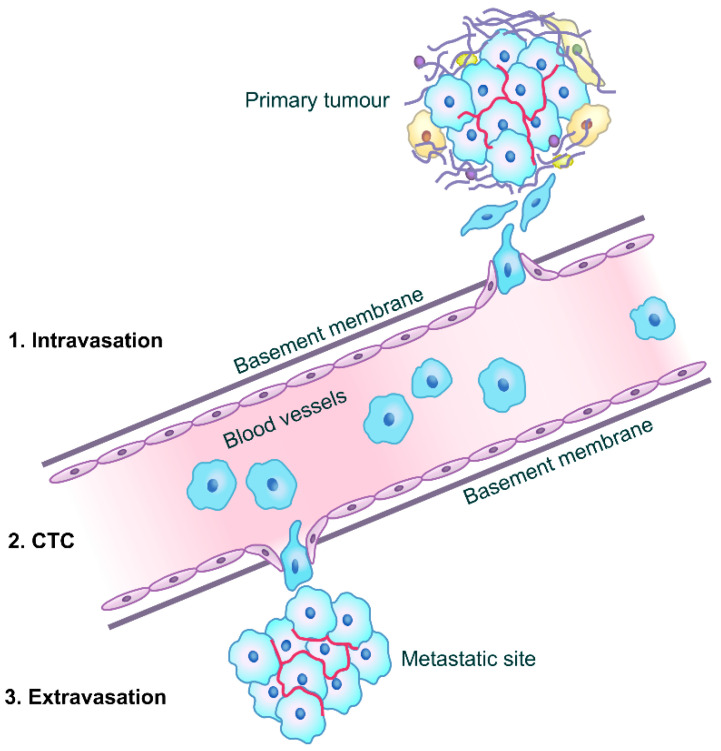
Diagrammatic representation of the metastatic cascade showing the three stages, namely, intravasation of primary tumor cells, before circulating in the bloodstream as CTCs, and extravasation at a distant site giving rise to a metastatic deposit [12,16].

The process of metastasis begins with intravasation (as shown in Figure 1), where the tumor cells remodel the cytoskeleton to facilitate migration through actin remodeling. The extracellular matrix is degraded by matrix metalloproteinases (MMPs) [11]. The cancer cell(s) then dissociate from the primary tumor, commonly via an epithelial to mesenchymal transition (EMT). EMT causes a decreased cell–cell contact, loosened associations, and cells to attain migratory properties [12], in the process lowering the expression of E-cadherin and increasing the expression of mesenchymal markers [13,14]. Cells, which then enter into blood vessels, are named circulating tumor cells (CTCs) and travel to the metastatic site. Lastly, extravasation occurs when cells form a pre-metastatic niche and alter the environment of the destination organ [15].

Breast cancer metastasis is still a major clinical problem. Despite the availability of several therapeutic modalities, including chemotherapy, endocrine therapy, immunotherapy and targeted therapy, treatment outcome remains poor (5-year overall survival rate < 30%) [3,17]. Hence, the efficacious treatment of early stage primary breast tumors and the prevention of metastatic spread, would significantly improve treatment outcome for breast cancer patients. In this regard, the concept of a precision medicine model where a group of breast cancer patients are selected based on a metastasis-related molecule which is subsequently, targeted (as elaborated in Section 5), seems to be an attractive therapeutic strategy. Precision medicine is an emerging approach for breast cancer therapy with the aim of selecting the optimal therapy for a specific group of cancer patients [18].

The discovery and characterization of molecules that are able to suppress metastasis holds great value in providing biological insights into mechanisms involved in this multifaceted process. It is, therefore, not surprising that metastatic suppressors are often reduced in expression in metastatic tumors, and upon overexpression or re-expression, are able to inhibit the metastatic ability of cancer cells; thus, slowing or preventing tumor spread. This review will focus on CD82, a metastatic suppressor, and evaluate its potential usage as a therapeutic strategy in overcoming the distant spread of breast cancer cells.

## 2. CD82 Glycoprotein

**Figure 2:**
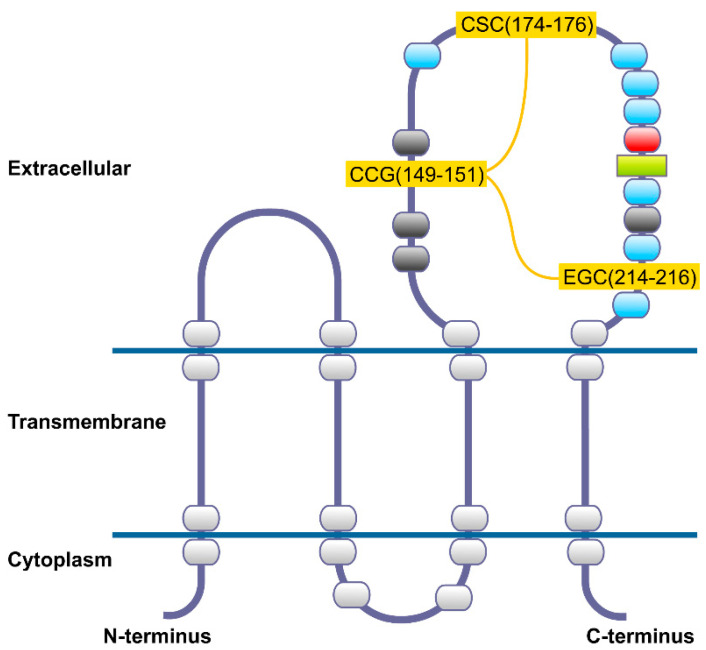
Schematic diagram of the CD82/KAI1 model showing the extracellular–transmembrane–cytoplasmic domains according to Bienstock and Barett [20]. Large extracellular domain contains cysteine protein motifs (CCG, CSC, and EGC) as yellow boxes with disulfide bonds shown as yellow lines. N-glycosylation sites are portrayed as dark grey boxes. The green rectangle represents another cysteine residue, red box depicts an asparagine residue and light blue boxes denote glutamine residues.

CD82, also known as KAI1 (kangai1–anticancer in Chinese), is a glycoprotein that belongs to the tetraspanin family. The *CD82* gene is located on human chromosome 11p11.2, and comprises 10 exons and 9 introns [19]. The CD82 protein is 267 amino acids long, with its predicted structure based on previously known tetraspanins, coupled with modelling of the individual sections [20] (Figure 2).

Tetraspanins are found on the cell membrane, and usually comprise four transmembrane domains, cytoplasmic N- and C-termini, a small and a large extracellular domain. The large extracellular domain contains asparagine residues that are N-glycosylated [21], and is divided into a constant region which contains α-helices, and a variable region which has cysteine residues that form intramolecular disulfide bonds [22]. The variable region is known to contain most of the known protein–protein interaction sites. Cysteine residues proximal to the membrane in the cytoplasmic domains are palmitoylated.

Each distinct domain of CD82 contributes to its overall activity. N-glycosylation is shown to play an essential role in the function of cell surface receptors and adhesion molecules [23,24]. Transmembrane polar residues have also been found to be important for the anti-cancer effects of CD82, including migration, invasion, metastasis, and membrane protrusion, as well as being involved in both the physical interactions within the lipid bilayer, and maintaining the overall conformation of the protein [25]. They also play an important role in intramolecular packing, and intermolecular interactions and interfaces [26]. Palmitoylation of the cysteine residues proximal to the membrane contributes to the localization of CD82 at the cell membrane, as well as its participation in the tetraspanin-enriched microdomain, contributing to its suppression of cell motility and invasion [27]. The small extracellular domain of CD82 has been found to be important for the anti-metastatic activity of CD82 [28]. CD82 anti-metastatic activity was observed to be lost upon the addition of negatively charged residues, or after perturbation of the secondary structure of the domain. The C-terminal of CD82 was discovered to play a significant role in its activity towards the epidermal growth factor receptor (EGFR). It was observed that upon the deletion of the C-terminus, the endocytic trafficking of CD82 was inhibited, which affected the regulation of the ligand-induced ubiquitylation of EGFR [29].

## 3. CD82 and Cancer Metastasis

CD82 is known to inhibit cell motility, which is important in the intravasation and extravasation steps in the metastatic cascade (as shown in Figure 1), and down-regulating cell–cell adhesion which would impede colonization at the metastatic site [30]. Some mechanisms have been proposed regarding how cell motility is facilitated by tetraspanins, such as the regulation of cell adhesion molecules or growth factor receptors, thereby altering cellular behavior [31]. In particular, CD82 attenuates cellular morphogenesis and EGFR signaling via downregulation of the associated integrin and EGFR [32,33]. The other possible mechanism involves tetraspanins’ cellular functions by signal initiation and transduction [31]. For instance, CD82 is known to diminish lamellipodia formation and perturb actin organization by the deregulation of Rac1, RhoA, and their effectors cofilin and Rho kinase. Tetraspanins have also been reported to control various cell functions such as cell adhesion, migration and communication via the regulation of digitation junctions [34]. CD82 has been observed to inhibit cell movement by hampering the formation and development of both cellular protrusions and retractions at the cellular level [31]. CD82 was also reported to attenuate the activation of β1 integrin and downregulate outside-in signaling [35], decreasing cell adhesion and motility. Knockdown of liprin α1 was observed to lead to an upregulation of CD82, inhibiting the formation of microprotrusions [25,36]. The CD82-mediated inhibition of tumor cell movement has been linked to its binding to cholesterol, and its coalescence with lipid rafts and tetraspanin-enriched microdomains [37].

Other aspects of the functional roles of CD82, include innate immune signaling through association with Toll-like receptor 9 (TLR9) in the endoplasmic reticulum (ER) and post-ER, and the modulation of the TLR9-dependent nuclear factor kappa-light-chain-enhancer of activated B cells (NF-κB) nuclear translocation [38].

## 4. Significance of CD82 Expression as a Metastasis Suppressor in Different Types of Cancer

### 4.1. Breast Cancer

CD82 was first reported as a potential marker for breast cancer metastasis by Yang et al. [39], who demonstrated that the metastatic propensity of a variety of breast cancer cell lines was inversely correlated with CD82/KAIl mRNA expression. Yang and co-workers further showed in another study that a lower CD82 protein expression was associated with breast malignancy as analyzed by immunohistochemistry in breast tissues from 81 patients (comprising 7 normal tissues, 7 ductal carcinoma in situ and 67 breast cancer tissues) [40]. CD82 mRNA expression was also observed to be reduced in breast cancer tissues as compared to normal breast tissue [41,42]. Malik et al. [42] further reported a significant correlation of the CD82 transcripts with TNM staging and that a higher expression of CD82 was associated with more favorable survival in breast cancer patients. In addition, CD82 mRNA expression was observed to be significantly reduced in breast cancer metastases to the brain [43]. CD82 protein expression detected by immunohistochemical staining, was reported to be significantly associated with the axillary lymph node status and advanced tumor stage, but no correlation was observed with the hormonal receptor (HR) or Human Epidermal Growth Factor- 2 (HER-2) receptor status [44,45]. In a study on 109 breast cancer patients, Huang et al. [46] observed that CD82-negative tumors had significantly lower 5-year disease-free survival compared to their CD82-positive counterparts; thus, showing that the reduction in CD82 expression is correlated with tumor recurrence. In another clinicopathological study, Christgen et al. [47] analyzed CD82 expression in 92 distant metastases from breast cancers, which included matched pairs of breast cancer and metachronous distant metastases. The same authors observed an association of CD82-positive metastases with the estrogen receptor (ER)-negative phenotype, implying that CD82 is not a good determinant of cancer progression in this breast cancer subtype. However, more in vitro and in vivo experimentation, as well as a larger cohort of breast cancer patients, are required to validate that metastasis in ER-negative tumors is independent of CD82.

Likewise, in vitro studies have revealed that CD82 plays an important role in cell adhesion, migration and invasion in breast cancer cells. The knockdown of CD82 expression in MDA-MB-231 breast cancer cells promoted cell migration and invasion, which was posited to be via the dysregulation of mitogen-activated protein kinase (MAPK) signaling, and the interaction of CD82 with EGFR [48,49]. In addition, CD82 was observed to inhibit cell adhesion which could possibly be mediated via its interaction with integrins [48]. The metastasis suppressive functions of CD82 have been reported to be abrogated by the splicing of the CD82 gene [50,51], where the CD82 spliced variant was observed to enhance cell migration and proliferation, concomitant with the activation of Src kinase in MDA-MB-231 breast cancer cells [50]. Overexpression of Sulfatase 2 (Sulf 2) was also reported to promote cell migration and invasion with the concomitant downregulation of CD82 expression in MDA-MB-231 breast cancer cells [52]. In the same study, Sulf 2 overexpressing MDA-MB-231 breast cancer cells were observed to invade surrounding muscle tissues, thereby demonstrating significant invasive ability in a mouse xenograft model.

Interestingly, the institution of endocrine therapy was successful in re-inducing the expression of CD82 in ER-positive breast cancer patients treated with ER antagonists [53]. In this same study, exposure to fulvestrant, a clinically approved ER antagonist was observed to upregulate CD82 expression in ER+ MCF-7 and T-47D breast cancer cells. In another clinically relevant study, Wang et al. [54] noted higher expression of CD82 in the serum exosomes from breast cancer patients, as compared with patients who had benign breast disease and healthy controls. They postulated that a redistribution of the CD82 protein via exosomes from breast cancer tissues to blood occurs during breast cancer development. Hence, expression levels of CD82 measured in exosomes could be useful as a potential biomarker for determining the metastatic potential in breast cancer.

### 4.2. Other Cancers

CD82 expression has also been reported in other cancer types, which include cancers of the prostate, lung and pancreas. CD82 was first discovered to be under-expressed in cell lines derived from metastatic prostate cancer cells in 1995 [55]. Another study further revealed that CD82 enhanced the shedding of E-cadherin through the suppression of disintegrin and metalloprotease 17 (ADAM17), while promoting motility, migratory, and invasive properties of prostate cancer cells [56]. CD82 is also known to inhibit cancer invasion and metastasis in non-small-cell lung carcinoma (NSCLC) via multiple mechanisms [57]. A higher expression of CD82 was reported in tumors which are better differentiated, less likely to metastasize to lymph nodes, and present at an earlier clinical stage in NSCLC [58]. In addition, the survival period of NSCLC patients with low CD82 was significantly shorter than patients with a positive expression of CD82. A reduced expression of CD82 has been reported in pancreatic cancer metastases [59,60]. A recent study showed that CD82 inhibits the EMT process in pancreatic cancer by increasing E-cadherin expression and reducing the expression of Snail, vimentin, MMP2, and MMP9, which are involved in different steps of the EMT process, thereby effectively reversing the EMT process [61]. A lowered expression of CD82 corresponding to increased differentiation associated with metastasis has been observed in cervical cancer [62]. Decreased CD82 expression was reported to be significantly correlated with advanced disease and poor prognosis in melanomas [63]. CD82 was also observed to be a potential and promising therapeutic target for acute myelogenous leukemia [64] and oral cancer [65]. Moreover, CD82 has been reported to inhibit invasion and metastasis of esophageal squamous cell carcinoma via the regulation of TGF-β1 [66], and decrease colon cancer cell motility [67].

The next section focuses on the therapeutic value of CD82 in curbing breast cancer metastasis, which is the cancer of interest in this review.

## 5. CD82 as a Therapeutic Target for Personalized Therapy in Breast Cancer

Currently, therapeutic strategies for breast cancer depend essentially on the classification of the breast cancer subtypes, *viz*., HR+/HER2−, HER2+ and triple-negative (HR−/HER2−) [68]. For non-metastatic breast tumors, local treatment involves surgery (which may entail the removal of axillary lymph nodes) or radiotherapy. Systemic therapy instituted would depend on the breast cancer subtype [68]. Basically, endocrine therapy (tamoxifen or aromatase inhibitors) is the mainstay of treatment for all HR+ tumors. Trastuzumab-based HER2 − directed monoclonal antibody therapy with chemotherapy is recommended for all HER2+ breast cancers (together with endocrine therapy in the presence of concurrent HR positivity). For triple negative breast cancer, chemotherapy is advocated, for instance the combination of cyclophosphamide with a taxane (docetaxel) or an anthracycline (doxorubicin), or combination with methotrexate and 5 Fluorouracil. More recently, immunotherapy, such as use of the monoclonal antibodies, pembrolizumab which inhibits programmed cell death 1 (PD-1) and atezolizumab, an inhibitor of the programmed cell death ligand 1 (PD-L1) that releases the suppression of the PD-1/PD-L1–mediated immune response in patients with triple negative breast cancer, has shown promise [69].

Personalized treatment and precision medicine, tailored towards specific individuals and subgroups of selected patients, respectively, are gaining traction in oncology, where an emphasis is placed on the prevention of disease progression, and the treatment regime is selected to maximize the efficacy and minimize toxicity [70]. As CD82 is known to have anti-metastatic properties (as elaborated in Section 4.1), this glycoprotein would be a good and suitable potential molecular target to be further explored for precision therapy in preventing breast cancer progression, when used in combination with systemic therapy according to the cancer subtype (as described in the above paragraph).

### 5.1. Selection of Suitable Breast Cancer Patients for Therapy

**Figure 3:**
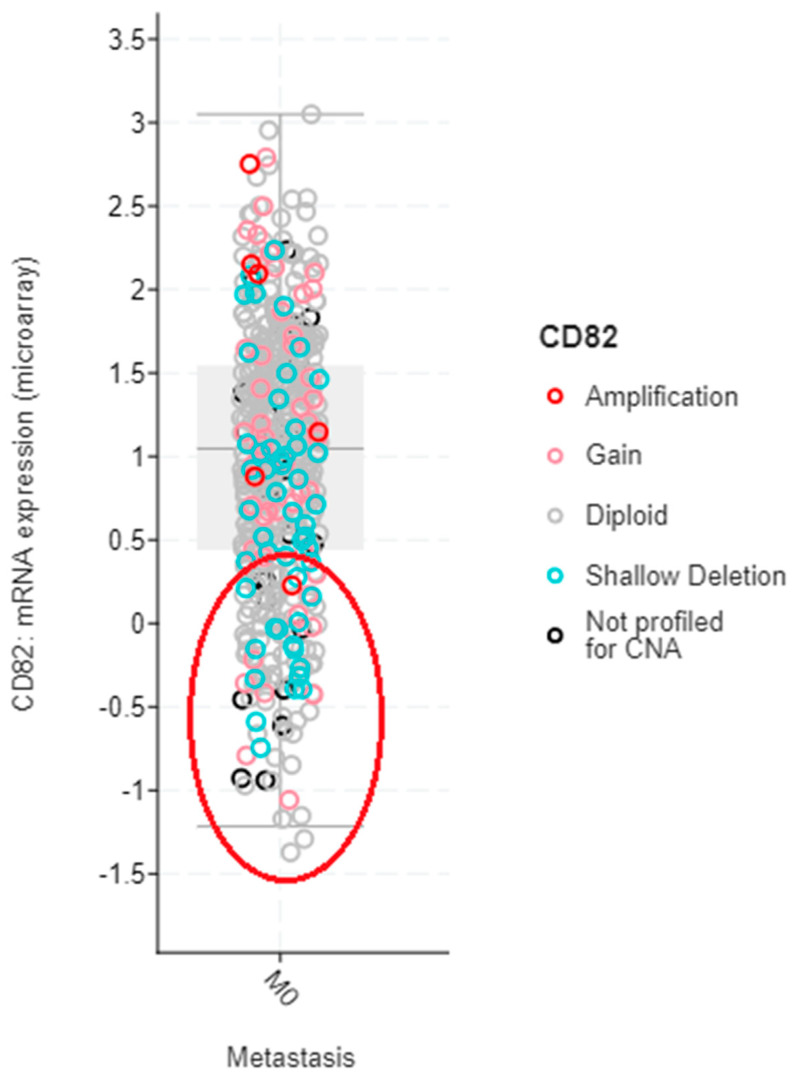
Box plot showing expression of CD82 in primary breast cancer tissues (*n* = 519) as derived from the cBioPortal database [72,73]. Datasets in the cBioPortal database are obtained from multiple sources, including Memorial Sloan Kettering Cancer Centre (MSKCC), Duke University–National University of Singapore (Duke-NUS), Samsung Medical Centre (SMC) and The Cancer Genome Atlas (TCGA), among others. The encircled region (delineated by the red oval) represents breast cancer cases with low expression of CD82.

Stage I-III non-metastatic breast cancer patients with a low expression of CD82 (such as those shown in Figure 3 below) can be first identified from patient biopsies, or potentially, exosomes from the bloodstream, which is an example of a liquid biopsy using a non-invasive approach [71]. Selected patients could then undergo targeted therapy to restore CD82 in order to minimize the possibility of metastasis.

### 5.2. Potential Therapeutic Options for Upregulating CD82 in Breast Cancer

#### 5.2.1. Drugs Known to Target CD82

##### Tyrosine Kinase Inhibitors (TKIs)

**Figure 4:**
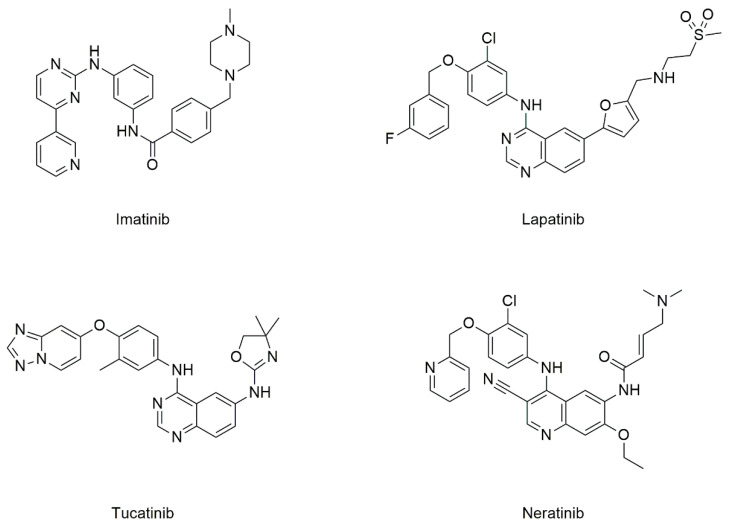
Chemical structures of imatinib, lapatinib, neratinib, and tucatinib.

Imatinib, a Type 2A TKI [74] (Figure 4), has been widely used in chemotherapy for chronic myeloid leukemia (CML), among others [75]. Imatinib was the first kinase inhibitor to be approved by the FDA in 2001 [76]. Additionally known as “Gleevec” or “Glivec”, imatinib was once hailed as the “magical bullet” that could cure cancer [77]. Although imatinib is known to inhibit Abelson (ABL) tyrosine kinase, which is expressed in CML, its ‘polypharmacology’ has facilitated its use in the therapy of several types of cancers [78], such as gastrointestinal stromal tumors [79]. However, imatinib when used singly, was found to lack clinical activity in Platelet-Derived Growth Factor Receptor-overexpressing metastatic breast cancer (MBC), with potential immunosuppressive effects [77]. Interestingly, it was reported that imatinib up-regulated *CD82* gene expression in human MCF-7 breast cancer cells, concomitant with a significant inhibition in cell proliferation [80]. However, there has not been much follow-up to explore its use for preventing breast cancer metastasis.

TKIs such as lapatinib, neratinib, and tucatinib (Figure 4), which are HER-2-specific, have demonstrated efficacy in the management of MBC [81,82]. In fact, lapatinib was the first FDA approved TKI to treat HER2 − positive MBC in combination with capecitabine [83], with neratinib being approved later on in 2020 for the same purpose [84]. Neratinib has also been reported to be effective in treating early HER-2+ breast cancers as shown by the results from the ExteNET trial [85]. Furthermore, tucatinib, yet another selective HER2 inhibitor, was also approved in 2020 for breast cancer patients who developed brain metastases [86,87].

Currently, unlike imatinib, it is not known if lapatinib, neratinib, and tucatinib have the propensity to upregulate CD82 expression. It would be worthwhile to investigate if the latter three drugs have any effect on CD82 expression, so as to explore the possibility of repositioning these TKIs for treating primary breast cancers with the goal of eradicating metastasis.

##### Etoposide

**Figure 5:**
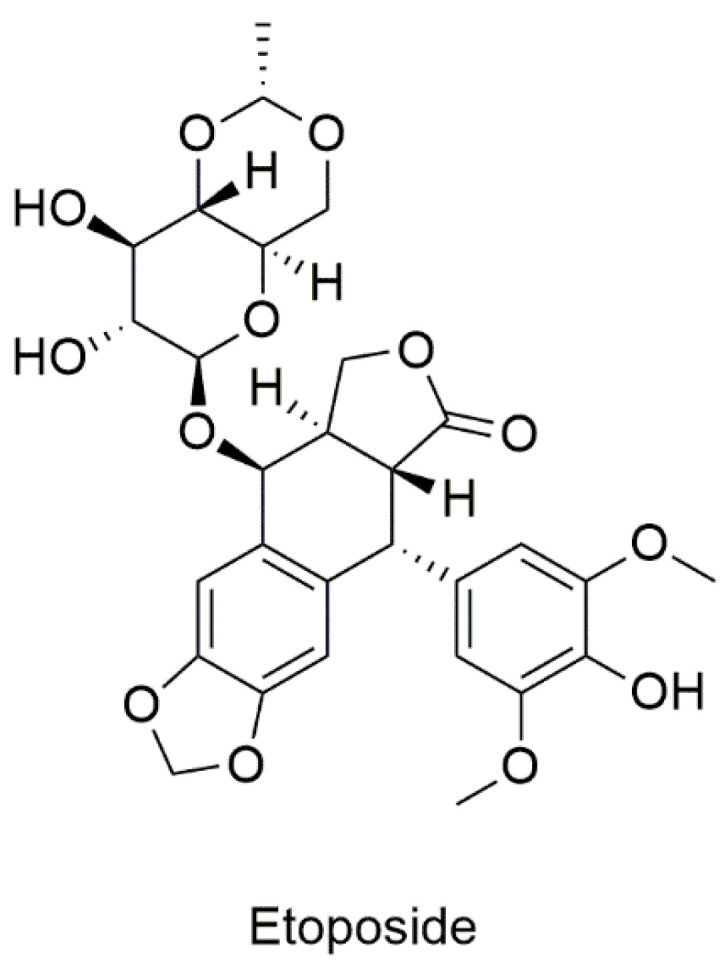
Chemical structure of etoposide.

DNA topoisomerases (categorized into DNA topoisomerase I and DNA topoisomerase II) are enzymes that play essential roles in DNA replication and transcription. Etoposide, a podophyllotoxin derivative (Figure 5), is a topoisomerase II inhibitor which was discovered to have increased antineoplastic activity and synthesized as etoposide (VP-16) in 1966 [88]. Since then, etoposide has been used in the treatment of small cell lung cancer, lymphomas, ovarian cancer, and breast cancer.

The results for treatment of metastatic cancers using etoposide in combinational therapy have been promising for some metastatic cancers but mixed for MBC. Oral etoposide was assessed as a valuable and safe option for pre-treated MBC patients [89,90]. Moreover, etoposide with apatinib has been reported to be effective and tolerable in heavily pretreated, metastatic HER2 − negative breast cancer patients [91]. On the other hand, etoposide in combination with irinotecan in a Phase II trial for refractory MBC, was terminated as the interim analysis revealed severe toxicity effects [92]. In fact, two decades ago, it was also reported that a chronic oral regimen of etoposide for 21 days produced significant toxicity [93].

Interestingly, etoposide has been previously observed to activate CD82 in a dose-dependent manner via p53 and c-Jun, in human prostate cancer cell lines [94]. Moreover, etoposide has been shown in breast cancer cell lines to induce p53 expression which could then upregulate CD82 expression [95,96]. Thus, it may be meaningful to verify if etoposide upregulates CD82 expression in breast cancer patients, for repurposing as a therapeutic agent in treating a subset of non-metastatic breast cancer patients with low CD82 expression. This is especially since etoposide at a low dose is preferred by some clinicians because of its relatively lower cost compared to other chemotherapeutic drugs and oral route of administration (thereby avoiding visits to the clinic for drug infusions) [97].

#### 5.2.2. CD82 Mimics

Instead of targeting CD82, attempting to mimic the activity of CD82 could be another potential method of promoting the metastatic suppressing ability of CD82. Targeting protein–protein interactions that involve CD82 is a potential method of CD82 activation. Recombinant soluble long extracellular loops (LELs) mimicking the CD82 LEL (present in its transmembrane domain as shown in Figure 2), could be a possible approach for activating pathways that involve CD82. Recombinant LELs have been utilized to inhibit infection of macrophages in HIV [98]. Recently, a peptide mimicking the small extracellular domain (EC1) of CD82 has been used to treat various cancer cells, including colon, breast, prostate, and lung cancer cells [99]. In this same study, the EC1 amino acid sequence mimic peptide of CD82 (CD82EC1-mP) was successful in inhibiting cell migration, invasion and adhesion in vitro, while also suppressing metastasis in lung cancer cells in mice. With respect to breast cancer, CD82EC1-mP was observed to enhance homotypic cell–cell aggregation and inhibit the cell migration and invasion in MDA-MB-231 breast cancer cells. Further mechanistic studies suggested that the suppression of metastasis was mediated via the inhibition of the EMT process through the modulation of the Wnt and Hippo pathways [100].

#### 5.2.3. Epigenetic Drugs for Treatment of Breast Cancer

##### Long Non-Coding RNA (lncRNA)-Based Therapy

lncRNAs are RNAs more than 200 nucleotides in length with a functional significance in transcriptional and post-transcriptional silencing [101]. Recently, a novel lncRNA named SKAI1BC (Suppressor of KAI1 in Breast Carcinoma) has been shown to epigenetically inactivate the anti-oncogenic activity of KAI/CD82 in breast cancer [102]. Hence, SKAI1BC could be a potential target for lncRNA-based therapy in preventing metastatic spread in breast cancer. The approach taken towards inhibiting oncogenic lncRNA in breast cancer, thus far, includes the use of antisense oligonucleotides, treatment with locked nucleic acids (DNA analogues), and nanoparticle-mediated RNA interference (RNAi) technology [103].

##### MicroRNA (miRNA) Therapeutics

MicroRNAs (miRNAs) are small RNA molecules that are involved in the regulation of gene expression [104] by inducing mRNA degradation and repressing translation in cells. Recently, studies on miRNA therapeutics have shown that miRNA delivery can be useful in treating diseases [105]. Although rarer, translational upregulation by miRNAs has been observed either through direct activation via miRNA/miRNP, or the relief of repression, where the activity of a repressive miRNA or miRNP is negated [106]. miRNA therapy, touted as new generation therapeutics [107], can be classified into oncogenic miRNA inhibition, and tumor-suppressor miRNA mimics [108].

Tumor-suppressive miRNAs tend to be under-expressed in breast cancers [109]. For instance, miR-124 expression was reported to be lower in higher grade breast tumor tissues and highly metastatic MDA-MB-231 cell line [110]. Further studies have revealed that miR-124 could inhibit breast cancer invasion, and metastasis [111,112]. miR-125a-5p was significantly downregulated in breast cancer, concomitant with a lower overall free survival and progression-free survival [113], and observed to suppress breast cancer cell proliferation and migration [114]. Interestingly, miR-125a-5p was able to overcome chemoresistance when paired with chemotherapeutic drugs [115]. miR-137, miR-139, and miR-145 were reported to be significantly downregulated in triple negative breast cancer and have been shown to be effective in increasing the susceptibility of breast cancer to chemotherapy, in addition to inhibiting cell proliferation and metastasis [116,117,118,119,120,121]. miR-671-5p was observed to suppress cell proliferation and invasion, while sensitizing cells to radiotherapy [122,123]. On the other hand, oncogenic miRNAs are usually overexpressed in breast cancers [109]. miR-96 promotes cell proliferation, migration, and invasion [124], while hindering apoptosis and drug resistance [125]. Additionally, miR-370 was noted to enhance metastasis and cell invasion [126,127].

To date, there have been no reports in the literature on miRNAs that target CD82 in breast cancer. Interestingly, a search, using the miRWalk database [128], revealed that there are close to 500 miRNAs that are predicted to regulate the expression of the CD82 gene. In fact, all the miRNAs associated with breast cancer that were mentioned in the above paragraph have been predicted by the miRWalk database to regulate CD82 mRNA expression. With regard to other cancer types, miRNAs have also been observed to promote cancer metastasis via their modulation of CD82 expression in hepatocellular cancer, malignant melanoma and gastric cancer [129,130,131,132,133].

As CD82 is a metastasis suppressor, oncogenic miRNAs which reduce its expression are up-regulated during the metastatic process, and can be targeted by the delivery of miRNA antagonists that are complementary to the targeted miRNA or decoying the target miRNA with sponge RNAs [134]. The general steps in using this strategy would involve the selection of the miRNA candidate, the validation of its presence in a patient sample, designing the anti-miRNA inhibitor and developing a safe and effective delivery system [107]. Although many miRNA-based therapies have been explored in breast cancer, research and trials concerning their clinical applications are still in their early stages, and more work needs to be conducted before miRNAs could become translatable in clinical practice as breast cancer therapeutics [135]. Nonetheless, miRNAs remain as promising targets due to the wide range of pathways that they can affect and their ability to enhance the effects of current chemotherapeutic drugs.

## 6. Challenges and Future Directions

The fact that breast cancer harbors molecular and cellular heterogeneity poses a considerable challenge in the attempt to unravel the complex relationship between the molecular biology of cancer and response to a specific therapeutic strategy, since there could be many drivers leading to cancer progression [3,136]. Hence, the need for innovative approaches such as precision oncology, especially in the case of triple-negative breast cancer which is known to be a very heterogeneous subset of breast cancer [137].

The proposed TKIs and etoposide in Section 5.2.1 are all FDA-approved drugs for cancer treatment. The repurposing of existing drugs would, therefore, be especially beneficial for treating triple-negative breast cancer patients, as there is a lack of targeted therapies for this subtype of breast cancer. It is anticipated that with advancements in computational methods related to chemoinformatics and genomics, more existing (both approved and investigational) drugs would be repositioned to treat breast cancer [138].

The development of CD82 mimics for therapy is still in the infancy stage. As the CD82 EC1 mimic is a natural peptide, it is unlikely to exert any immunogenic effects [99,139]. However, the flip side is that since the CD82 protein is also present in normal tissues, it is essential to ascertain the safety profile of the peptide in the human body before clinical use. More preclinical investigations and clinical trials are needed to establish the optimal therapeutic window for the efficient and safe clinical application of such mimetics.

For non-coding RNA-based therapeutics, the litmus test is to be able to identify the most efficacious lncRNA and miRNA candidates with good safety profiles and achieve an effective targeted delivery to the breast cancer tissues. In terms of miRNA therapeutics, the surge in the availability of genomic and proteomic data would facilitate the identification of major miRNA targets [107], which together with the recent development of efficient therapeutic miRNA delivery systems [140], could enhance miRNA-based therapeutics for cancer.

Although the prevalent view used to be that tumor suppressors (which include metastasis suppressors) are un-targetable [141], there is now a paradigm shift since the recent report of Hsiue et al. [142] on p53 mutant peptide-targeted immunotherapy, albeit an antibody-based therapeutic that targets the most commonly mutated tumor suppressor gene.

## 7. Conclusions

CD82 is known to play a significant anti-metastatic role in multiple cancers, including breast cancer. Currently, treatment strategies involving CD82 as a therapeutic target to hinder breast cancer spread has not been fully exploited, even though potential drugs such as the TKIs, lapatinib, neratinib, and tucatinib have been approved for MBC treatment, while imatinib and etoposide have been investigated in clinical trials for MBC. The latter two drugs exhibit their anti-metastatic activity through the activation of CD82 via different pathways as mentioned earlier, while it is at present not known if the actions of lapatinib, neratinib, and tucatinib are also mediated in part via the CD82 glycoprotein. The re-profiling of these clinically approved drugs for treating non-metastatic breast cancer would also reduce patient safety issues, such as risks of adverse effects, and bring down the cost of treatment. Whether CD82 mimics or non-coding RNA therapeutics will be part of the arsenal in overcoming breast cancer metastasis remains to be further investigated. For precision therapeutics to be successfully implemented in breast cancer patients, clinical oncologists must overcome the inertia to change standard practice and be prepared to conduct precision oncology-based clinical trials to validate the efficacy of novel therapeutic approaches [143,144].
